# The dilemma of 12/14F ureteral access sheath (UAS) usage: a case control study

**DOI:** 10.1186/s12894-022-01031-6

**Published:** 2022-06-15

**Authors:** Tomasz Ozimek, Judith R. Wiessmeyer, Julian P. Struck, Marie C. Roesch, Nils Gilbert, Jan M. Laturnus, Axel S. Merseburger, Mario W. Kramer

**Affiliations:** Department of Urology, University Hospital Schleswig-Holstein, Campus Lübeck, Ratzeburger Allee 160, 23562 Lübeck, Germany

**Keywords:** Flexible ureteroscopy, Ureteral access sheath, Kidney stones, Urolithiasis, Complications

## Abstract

**Background:**

The insertion of a ureteral access sheath (UAS) is a frequent procedure during flexible ureteroscopy (fURS) to facilitate kidney stone treatment. The aim of this study was to investigate the influence of 12/14 French (F) UAS on fURS outcomes.

**Methods:**

We performed a retrospective monocentric analysis of fURS procedures conducted at the Department of Urology (University Hospital Schleswig–Holstein, Lübeck, Germany) for kidney stone treatment via lithotripsy or basket stone retrieval between September 2013 and June 2017. Uni- and multivariate analyses were done with the help of RStudio (Version 1.0.136) software.

**Results:**

In total, 283 consecutive fURS were analyzed. UAS was applied in 98 cases (34.63%). The insertion of UAS was preferred in cases with multiple kidney stones and larger median maximal stone diameter (*p* < 0.05). UAS usage correlated with elevated radiation exposure in seconds (94 vs. 61; *p* < 0.0001), prolonged operation time in minutes (99 vs. 66, *p* < 0.0001), length of hospital stay over 48 h (LOS, 22.49% vs. 10.81%; *p* = 0.015), more frequent postoperative systemic inflammatory response syndrome (SIRS, 13.27% vs. 4.32%; *p* = 0.013) and lower postoperative stone-free rates (60.20% vs. 78.92%; *p* = 0.0013). Moreover, we conducted uni- and multivariate subgroup analysis for cases with multiple kidney stones (≥ 2) and comparable stone burden; UAS was inserted in 48.3% of these cases (71/147). On multivariate logistic regression, UAS insertion was statistically associated with prolonged operation time in minutes (101 vs. 77; *p* = 0.004). No statistical differences regarding radiation exposure, stone-free rates, postoperative SIRS rates or LOS were noted between UAS and non-UAS patients with multiple kidney stones of similar size (p > 0.05).

**Conclusions:**

12/14F UAS does not seem to improve overall outcomes in fURS for kidney stones. In patients with multiple kidney stones it may be associated with elevated operation time without a clear benefit in terms of improved stone-free status or reduced perioperative complication rate. Further prospective randomized studies to specify the indications for UAS usage are urgently needed.

## Background

The insertion of a ureteral access sheath (UAS) is a frequent procedure during flexible ureteroscopy (fURS) for kidney stone treatment [1]. Decreased intrarenal pressure as well as better irrigation and visibility are the rationale for UAS usage, to provide more efficient, faster and safer stone retrieval [2–4]. However, the current scientific evidence delivers many dilemmas that should be contemplated by the endourologist before UAS insertion. Firstly, reduced intrarenal pressure provided by the UAS seems to not result in a lower risk of postoperative complications, as numerous groups have reported either no effect or even an elevated risk of postoperative complications associated with UAS usage [5–7]. Moreover, the direct impact of UAS on the improvement of stone-free rates is arguable, according to the available literature [8–10]. The guidelines of the European Association of Urology (EAU) give no recommendation on UAS usage, whereas the American Urological Association advocates for UAS usage for cases with a complex stone burden [11, 12]. Based on the aforementioned scientific background, the aim of our study was to retrospectively investigate the influence of 12/14 French (F) UAS on fURS outcomes at our department and to discuss the future directions for research on UAS.

## Methods

A retrospective monocentric analysis of fURS procedures was performed, conducted at the Department of Urology (University Hospital Schleswig–Holstein, Lübeck, Germany) for kidney stone treatment via lithotripsy or basket stone retrieval between September 2013 and June 2017.

The details of the fURS procedure have already been described in our previous manuscripts [13, 14]. The patients consented to fURS at least 24 h preoperatively. Urine culture was gathered less than 14 days before the surgery. In cases with positive preoperative urine culture, antibiotic therapy was begun, usually at least 2 days preoperatively. Antibiotic prophylaxis was defined as a preoperative or intraoperative administration of antibiotics in cases with negative preoperative urine culture. Antibiotic prophylaxis was applied dependent on the decision of the endourologist. All fURS procedures were conducted in a supine lithotomy position. The surgeries were performed with reusable flexible ureteroscopes: digital Olympus URF-V or fiberoptic Karl Storz Flex-X2. fURS in our department is usually preceded by semirigid ureteroscopy (URS). Prior to the use of fURS, contrast (Urolux Retro®, CS Diagnostics GmbH) was injected into the proximal ureter via semirigid ureteroscope to present the pyelocalyceal anatomy. Ureteral access sheath (Olympus UroPass; 12/14F) insertion was not a standard in every fURS procedure. The decision was based on the preference of the surgeon, which depended mostly on the extent of the stone burden.

Holmium laserlithotripsy (Lumenis VersaPulse® PowerSuite™ 100 W) was performed with a reusable SlimLine™ 200 μm fiber (Boston Scientific). Total laser energy was defined as the cumulative lithotripsy energy via the semirigid and flexible ureteroscope. Calculi extraction during fURS was conducted with Stonizer® tipless (1.9F, Uromed) or NGage® (2.2F, Cook Medical) nitinol baskets. A ureteral double-J stent was reinserted depending on the complexity of fURS and significance of ureteral lesions.

Stone-free status (SFS) was determined intraoperatively by the endourologist with the assistance of intraoperative fluoroscopy. Radiological postoperative re-evaluation with computed tomography (CT) or delayed kidney, ureter and bladder (KUB) radiography was always performed in case of uncertainty regarding SFS. The length of hospital stay (LOS) for a routine postoperative course was limited to 48 postoperative hours. Every patient with at least one stone episode in the past was categorized as a recurrent stone former.

The determination of the infundibulopelvic angle (IPA) was based on preoperative native CT images in the coronal plane and intraoperative retrograde pyelography (RPG) images. The IPA was measured in accordance with the El-Bahnasy definition, as the angle between the ureteropelvic axis and the central axis of the lower pole of the infundibulum [15]. CT-based IPA was measured only in patients with a reliable outline of the proximal ureter and lower calyx on coronal CT images.

Intra- and postoperative complications were classified according to the Clavien-Dindo scale [16]. Following the definition of systemic inflammatory response syndrome (SIRS), at least two out of four criteria (body temperature < 36 °C or > 38 °C, heart rate > 90 bpm, respiratory rate > 20 per minute, WBC < 4000 or > 12,000 cells/mm^3^) were fulfilled in patients assigned to postoperative SIRS group [17].

Uni- and multivariate analyses were conducted with the help of RStudio (Version 1.0.136) software. The descriptive statistics comprise the mean value with standard deviation (SD) for normally distributed data, the median value with interquartile range (IQR) for data without a normal distribution, and percent values for categorical data. The chi^2^ test was applied for categorical data whenever applicable. The normal distribution of quantitative data was tested with the Shapiro–Wilk test. Student’s t-test was conducted for univariate analysis of the variables with a normal distribution. The parameters without a normal distribution were analyzed with the Mann Whitney-U test (MWU). The level of significance was *p* < 0.05. The present study obtained approval from the ethical committee of the University of Lübeck.

## Results

In total, 283 consecutive fURS for stone surgery performed between September 2013 and June 2017 were analyzed. 12/14F UAS was applied in 98 cases (34.63%). On univariate analysis, the insertion of 12/14F UAS was preferred in cases with multiple kidney stones [UAS: 71/98 (72.45%) vs. non-UAS: 76/185 (41.08%); *p* < 0.0001] and larger median maximal stone diameter [UAS: 7 mm (IQR 5; 10) vs. non-UAS: 6 mm (IQR 4; 9); *p* = 0.042]. Detailed results of univariate testing are presented in Table 1. Multivariate binary logistic regression confirmed the overall statistical correlation of 12/14F UAS usage with elevated radiation exposure [UAS: 94 s (IQR 67; 142) vs. non-UAS: 61 s (IQR 33; 100); *p* < 0.0001], prolonged operation time [UAS: 99 min (IQR 76; 121) vs. non-UAS: 66 min (IQR 46; 91), *p* < 0.0001], length of hospital stay [LOS > 48 h; UAS: 22/98 (22.49%) vs. non-UAS: 20/185 (10.81%); *p* = 0.015], more frequent postoperative SIRS [UAS: 13/98 (13.27%) vs. non-UAS: 8/185 (4.32%); *p* = 0.013] and lower rate of postoperative SFS [UAS: 59/98 (60.20%) vs. non-UAS: 146/185 (78.92%); *p* = 0.0013]. Reported relevant complications were defined as at least Clavien-Dindo Grade 2 (CD ≥ 2) events. In the UAS group, these included one intraoperative bleeding with postoperative SIRS, one ureteral perforation with postoperative SIRS, and eleven postoperative SIRS without intraoperative complications. In the non-UAS group, we recorded two intraoperative perforations of the calyceal system, one ureteral perforation, eight postoperative SIRS without intraoperative complications, one relevant postoperative gross hematuria, and one aspiration pneumonia.

**Table 1:** Overall univariate analysis of 12/14F ureteral access sheath (UAS) usage

		Total	UAS	No UAS	*p *value	Test
General characteristics	Number of cases	283	98	185		
	Gender (male/female)	194/89	62/36	132/53	0.2079	Chi2
	Side (Right/Left)	124/283 (43.82%)	43/98 (43.88%)	81/185 (43.78%)	1	Chi2
	Median Age (IQR), years	56 (42; 68)	54 (38; 68)	58 (45; 68)	0.3604	MWU
	BMI (IQR)	27 (25; 31)	27 (25; 30)	27 (24; 31)	0.9827	MWU
	ASA > 2*	54/262 (20.61%)	23/91 (25.27%)	31/171 (18.12%)	0.2297	Chi2
	Recurrent Stone Former	119/283 (42.05%)	50/98 (51.02%)	69/185 (37.30%)	0.0359	Chi2
	Prestenting	268/283 (94.70%)	96/98 (97.96%)	172/185 (92.97%)	0.1330	Chi2
	Positive preoperative urine culture**	81/259 (31.27%)	42/90 (46.67%)	39/169 (23.08%)	0.0002	Chi2
	Antibiotic prophylaxis***	64/177 (83.12%)	24/48 (50.00%)	40/129 (31.01%)	0.0306	Chi2
Stone characteristics	Concomitant Ureterolithiasis	82/283 (28.96%)	28/98 (28.57%)	54/185 (29.19%)	1	Chi2
	Lower Pole Kidney Stone	213/283 (75.27%)	82/98 (83.67%)	131/185 (70.81%)	0.0250	Chi2
	Median Lower Pole Kidney Stone Diameter (IQR), mm	6 (4; 8)	6 (4; 9)	5 (5; 8)	0.4678	MWU
	Median Kidney Stone Max. Diameter (IQR), mm	6 (5; 9)	7 (5; 10)	6 (4; 9)	0.0420	MWU
	Multiple Kidney Stones (≥ 2)	147/283 (51.94%)	71/98 (72.45%)	76/185 (41.08%)	< 0.0001	Chi2
	Median HU (IQR)	900 (60; 1034)	900 (550; 1000)	900 (600; 1036)	0.3566	MWU
Operative characteristics	Median IPA (SD), degrees	54 (42; 64)	52 (40; 60)	56 (43; 66)	0.0497	MWU
	Median IPA – preoperative imaging (IQR), degrees	51 (40; 64)	44 (37; 60)	52 (41; 66)	0.0661	MWU
	Safety Wire****	220/274 (80.59%)	73/95 (76.84%)	147/178 (82.58%)	0.3261	Chi2
	Preceding semirigid URS	198/283 (69.96%)	63/98 (64.29%)	135/185 (72.97%)	0.1674	Chi2
	Stone Extraction with fURS	263/283 (92.93%)	94/98 (95.92%)	169/185 (91.35%)	0.2370	Chi2
	Postoperative Stenting	271/283 (95.76%)	98/98 (100.00%)	173/185 (93.51%)	0.0234	Chi2
	Median Fluoroscopy Time**** (IQR), s	72 (44; 120)	94 (67; 142)	61 (33; 100)	< 0.0001	MWU
	Median Operation Time (IQR), min	77 (51; 105)	99 (76; 121)	66 (46; 91)	< 0.0001	MWU
	Laser in Calyceal System	170/283 (60.07%)	63/98 (64.29%)	107/185 (57.84%)	0.2920	Chi2
	Median Total Laser Energy (IQR), kJ	1.19 (0.39; 2.33)	1.22 (0.42; 2.41)	1.16 (0.39; 2.27)	0.4177	MWU
Outcomes	LOS > 48 h	42/283 (14.84%)	22/98 (22.49%)	20/185 (10.81%)	0.0145	Chi2
	fURS Defect	29/283 (10.25%)	13/98 (13.27%)	16/185 (8.65%)	0.3113	Chi2
	Clavien Dindo ≥ 2	26/283 (9.19%)	13/98 (13.27%)	13/185 (7.03%)	0.1304	Chi2
	Postoperative SIRS	21/185 (11.35%)	13/98 (13.27%)	8/185 (4.32%)	0.0127	Chi2
	Stone-free status	205/283 (72.44%)	59/98 (60.20%)	146/185 (78.92%)	0.0013	Chi2

ASA—American Society of Anaesthesiology scale; fURS—flexible ureteroscopy; HU—Hounsfield units; IPA—infundibulopelvic angle; IQR—interquartile range; LOS—length of hospital stay; MWU—Mann Whitney-U test; SIRS—systemic inflammatory response syndrome; URS- ureteroscopy

*No data in 21 cases

**No data in 24 cases

***Only cases with negative preoperative urine culture included. No data in 1 case

****No data in 10 cases

In order to reduce bias due to heterogenic cohorts demonstrated in aforementioned overall analysis, we conducted uni- and multivariate subgroup analysis for cases with multiple kidney stones (≥ 2) that are primarily considered for 12/14F UAS usage in our department. In this subanalysis stone characteristics were comparable between the cohorts. 12/14F UAS was inserted in 48.3% of cases (71/147), irrespective of median maximal stone diameter, median stone density, presence and size of lower pole stones (p > 0.05). Full results of the univariate testing for this subgroup are presented in Table 2. On multivariate logistic regression, 12/14F UAS insertion was statistically associated in the analyzed subgroup with prolonged operation time [UAS: 101 min (IQR 76; 128) vs. non-UAS: 77 min (IQR 54; 104); *p* = 0.004]. No differences regarding radiation exposure, SFR, postoperative SIRS rates and LOS were noted between UAS and non-UAS cases with multiple kidney stones of similar size (p > 0.05). The results of the multivariate binary logistic regression for this subgroup are presented in Table 3.

**Table 2:** Univariate analysis of UAS usage in patients with multiple kidney stones (≥ 2)

		Total	UAS	No UAS	*p *value	Test
General characteristics	Number of cases with multiple kidney stones (≥ 2)	147	71	76		
	Gender (male/female)	100/47	45/26	55/21	0.3218	Chi2
	Side (Right/Left)	58/89	31/40	27/49	0.4011	Chi2
	Median Age (IQR), years	56 (41; 70)	56 (39; 69)	55 (45; 70)	0.3199	MWU
	BMI (IQR)	26 (24; 30)	26 (24; 30)	27 (24; 30)	0.5150	MWU
	ASA > 2*	30/136 (22.06%)	17/64 (26.56%)	13/72 (18.06%)	0.3236	Chi2
	Recurrent Stone Former	74/147 (50.34%)	40/71 (56.34%)	34/76 (44.74%)	0.2147	Chi2
	Prestenting	138/147 (93.88%)	70/71 (98.59%)	68/76 (89.47%)	0.0500	Chi2
	Positive preoperative urine culture**	47/130 (36.15%)	29/65 (44.61%)	18/65 (27.69%)	0.0679	Chi2
	Antibiotic prophylaxis***	44/83 (53.01%)	23/36 (63.89%)	21/47 (44.68%)	0.1296	Chi2
Stone characteristics	Concomitant Ureterolithiasis	45/147 (30.61%)	25/71 (35.21%)	20/76 (26.32%)	0.3220	Chi2
	Lower Pole Kidney Stone	129/147 (87.76%)	62/71 (87.32%)	67/76 (88.16%)	1	Chi2
	Median Lower Pole Kidney Stone Diameter (IQR), mm	5 (4; 8)	5 (4; 8)	6 (4; 8)	0.8806	MWU
	Median Kidney Stone Max. Diameter (IQR), mm	7 (5; 9)	7 (5; 9)	6 (5; 9)	0.8928	MWU
	Mean HU (SD)	855 (483)	811 (459)	905 (506)	0.1502	t-test
Operative characteristics	Mean IPA (SD), degrees	52.62 (17.26)	50.14 (17.13)	54.91 (17.06)	0.08612	t-test
	Median IPA – preoperative imaging (IQR), degrees	49 (41; 65)	44 (40; 60)	54 (43; 66)	0.07342	MWU
	Safety Wire****	114/143 (79.72%)	53/69 (76.81%)	61/74 (82.43%)	0.5305	Chi2
	Preceding semirigid URS	96/147 (65.31%)	44/71 (61.97%)	52/76 (68.42%)	0.5173	Chi2
	Stone Extraction with fURS	139/147 (94.56%)	70/71 (98.59%)	69/76 (90.79%)	0.08543	Chi2
	Postoperative Stenting	143/147 (97.29%)	71/71 (100.00%)	72/76 (94.74%)	0.1463	Chi2
	Median Fluoroscopy Time**** (IQR), s	85 (54; 139)	101 (65; 140)	71 (41; 128)	0.0188	MWU
	Median Operation Time (IQR), min	92 (58; 118)	101 (76; 128)	77 (54; 104)	0.0041	MWU
	Laser in Calyceal System	97/147 (65.99%)	43/71 (60.56%)	54/76 (71.05%)	0.2431	Chi2
	Median Total Laser Energy (IQR), kJ	1.24 (0.54; 2.41)	1.27 (0.88; 2.41)	1.04 (0.48; 2.35)	0.2599	MWU
Outcomes	LOS > 48 h	28/147 (19.05%)	17/71 (23.94%)	11/76 (14.47%)	0.2109	Chi2
	fURS Defect	18/147 (12.24%)	11/71 (15.49%)	7/76 (9.21%)	0.3631	Chi2
	Clavien Dindo ≥ 2	16/147 (10.88%)	10/71 (14.08%)	6/76 (7.89%)	0.3477	Chi2
	Postoperative SIRS	13/147 (8.84%)	10/71 (14.08%)	3/76 (3.95%)	0.0611	Chi2
	Stone-free status	95/147 (64.63%)	42/71 (59.15%)	53/76 (69.74%)	0.2427	Chi2

ASA—American Society of Anaesthesiology scale; fURS—flexible ureteroscopy; HU—Hounsfield units; IPA – infundibulopelvic angle; IQR—interquartile range; LOS—length of hospital stay; MWU—Mann Whitney-U test; SIRS—systemic inflammatory response syndrome; URS- ureteroscopy

*No data in 11 cases

**No data in 17 cases

***Only cases with negative preoperative urine culture included

****No data in 4 cases

**Table 3:** Binary logistic regression of UAS usage in patients with multiple kidney stones (≥ 2)

	Odds ratio	95% confidence interval	*p* value
Positive preoperative urine culture**	1.2113	0.4748–3.0927	0.6861
Lower pole kidney stone diameter	0.9856	0.7718–1.2685	0.9077
Kidney stone max. diameter	0.9602	0.7393–1.2222	0.7474
Fluoroscopy time****	0.9948	0.9869–1.0009	0.1382
Operation time	1.0230	1.0100–1.0379	0.0009
LOS > 48 h	3.3989	0.8420–15.0767	0.0911
fURS defect	4.6702	0.9356–35.8181	0.0860
Postoperative SIRS	0.3997	0.0397–4.6656	0.4365
Stone-free status	0.5909	0.1793–1.8851	0.3754

fURS—flexible ureteroscopy; LOS—length of hospital stay; SIRS—systemic inflammatory response syndrome

**No data in 17 cases

****No data in 4 cases

## Discussion

Our study presents the retrospective outcomes of 12/14F UAS usage during fURS. Overall analysis as well as subanalysis for multiple stones of similar burden confirmed no clear benefit of 12/14F UAS. Due to the heterogeneity of stone characteristics demonstrated in overall analysis we suggest a special focus on the subgroup analysis of statistically comparable cases with multiple kidney stones (≥ 2) and similar stone size.

The reduction of bacterial backflow to the venous system due to lowered intrarenal pressure during ureteroscopy has been proposed as a rationale for UAS usage in sepsis prevention [18, 19]. Adversely, we recorded a trend towards the development of SIRS in overall analysis of the patients operated on with the help of 12/14F UAS. Uneven rate of positive preoperative urine cultures between the groups has to be seen as a negative consequence of the retrospective character of the study. The trend towards perioperative SIRS in UAS patients was rejected by subanalysis for multiple calculi cohort, where general and stone characteristics as well as microbiologic urine status between the UAS and non-UAS groups where statistically comparable. However, a protective role of UAS usage regarding SIRS could not be confirmed.

Moreover, we were unable to prove the benefit of 12/14F UAS usage on SFS, fluoroscopy and operating time neither in the overall cohort, nor in subgroup analysis of cases with multiple calculi.

The analyzed cases operated with UAS were correlated in uni- and multivariate analysis with a prolonged operating time. We should discuss the possible reasons of these unexpected results. A relevant percentage (ca. 40%) of fURS is being performed at our department by residents in training [14]. fURS with UAS is in our opinion more challenging due to insertion-related issues, less intuitive orientation in calyceal system in a low-pressure environment and continuous attention to a correct position of UAS during repeated stone extractions. These factors, encountered especially by less experienced surgeons, could result in prolonged operative time of UAS group.

The presented results put into question the rationale for 12/14F UAS usage, but however should be cautiously discussed, taking into consideration the limitations of the study. Firstly, the usage of UAS was allowed in every case, depended on subjective surgeon preference. Despite the similar parameters characterizing both groups (UAS and non-UAS) with multiple calculi, we are aware that endourologists at our department tend to use UAS in patients with a more demanding anatomy or stone burden. Easier cases, even with multiple kidney stones, are often treated by fURS without UAS usage. This must be considered as a bias, diminishing the accuracy of the presented comparison.

Secondly, we acknowledge some deficits in gathered data due to the retrospective design of our study. The surgeries were performed mostly as “basketing” fURS for smaller stones without the need of lithotripsy, or “dusting plus basketing” versus “dusting plus active hand irrigation” fURS for larger stone burden. The exact type of the stone removal following the lithotripsy ( “dusting plus basketing” vs. “dusting plus active hand irrigation”) was not recorded in the study; hence our retrospective analysis does not provide data regarding how many UAS cases were conducted with active calyceal irrigation with a manual syringe pump. In our practice, some unexperienced surgeons may tend to irrigate more vigorously during fURS to obtain a clear view, which may possibly lead to infectious complications. Here UAS size plays a crucial role in lowering the pressure, especially during active irrigation, which may provide benefit especially in terms of preventing SIRS development. Noureldin et al. showed in a porcine in vivo model that 12/14 F UAS provided a sufficient, 12-fold reduction in intrarenal pressure during fURS with gravity irrigation, but only a fivefold reduction under active pump irrigation [4]. The best possible pressure reduction was obtained solely by 14/16F UAS – 1 cmH_2_O both under passive and active irrigation.

Thirdly, we were unable to record the detailed status of intraoperative ureteral lesions during fURS. The rate of ureteral perforations was low (one case for the UAS group and one case for the non-UAS group). The high rates of postoperative stenting in the analyzed cohort have to be interpreted as a preventive measure. Placement of a mono-J catheter or double-J catheter on the string should be discussed as an alternative for postoperatively stone-free patients to spare them additional cystoscopy for double-J stent extraction [20].

Despite the mentioned drawbacks, mostly due to the retrospective character, our study enriches the limited and ambiguous evidence on UAS application. Only one randomized controlled trial has been conducted so far on UAS insertion for kidney stone treatment. Kourambas et al. found no influence of 12/14F UAS usage regarding SFS and complications, but UAS application allowed for a significant reduction in operating time [21].

Traxer et al. confirmed in a multicentric prospective non-randomized study on a large number (2,239) of cases no influence of UAS on SFS as well as parallel significant reduction of infectious complications [8]. No subgroup analysis of different UAS sizes, as well as the non-randomized character of the study should be mentioned as the limitations of this study.

One of the few studies demonstrating an actual advantage of UAS usage in terms of higher rates of SFS was published by L’esperance et al. [9]. The authors presented retrospective results, where UAS application statistically improved SFS (79% vs. 67%, *p* = 0.042).

The literature describing differences between various UAS sizes is scarce. A broad majority of the cited publications analyzed the application of universal 12/14F UAS. 12/14F UAS is considered an “all-rounder”, eligible both for a stented and non-stented ureter [22]. Taking into consideration the extremely rare 12/14F UAS insertion-related complications in our study, further studies focusing on the outcomes of the larger 14/16F UAS are warranted in our opinion. The insertion of 14/16F UAS in patients may result in better stone removal and a reduction in intrarenal pressure and associated SIRS. We advocate for a prospective randomized trial of different sizes of UAS, standardized regarding the experience status of the surgeons, to reliably investigate the general benefit of UAS and to determine the specific indications for a given UAS size, taking into consideration the possible complications of UAS associated with mechanical traction and overstretching of the ureter [23, 24].

## Conclusion

Based on our retrospective results, the usage of 12/14F UAS does not seem to improve outcomes in fURS for kidney stones. In patients with multiple kidney stones it may be associated with elevated operation time without a clear benefit in terms of improved SFS or reduced perioperative complication rate. However, relevant limitations of retrospective study design have to be considered. Prospective randomized studies are urgently needed to define the conditions of effective, safe and cost-effective UAS usage.

## Data Availability

The datasets used and/or analyzed during the current study are available from the corresponding author on reasonable request.

## Abbreviations

ASA: American society of anaesthesiology scale
CD: Clavien-Dindo
CT: Computed tomography
EAU: European association of urology
F: French
fURS: Flexible ureteroscopy
HU: Hounsfield units
IQR: Interquartile range
KUB: Kidney, ureter and bladder radiography
LOS: Length of hospital stay
MWU: Mann Whitney-U test
RPG: Retrograde pyelography
SD: Standard deviation
SFS: Stone-free status
SIRS: Systemic inflammatory response syndrome
UAS: Ureteral access sheath
URS: ureteroscopy
